# Whole-body and muscle responses to aerobic exercise training and withdrawal in ageing and COPD

**DOI:** 10.1183/13993003.01507-2021

**Published:** 2022-05-12

**Authors:** Lorna E. Latimer, Dumitru Constantin-Teodosiu, Bhavesh Popat, Despina Constantin, Linzy Houchen-Wolloff, Charlotte E. Bolton, Michael C. Steiner, Paul L. Greenhaff

**Affiliations:** 1 Dept of Respiratory Sciences, University of Leicester, Leicester, UK; 2 Institute for Lung Health, National Institute for Health Research Leicester Biomedical Research Centre – Respiratory, Glenfield Hospital, Leicester, UK; 3 MRC-Versus Arthritis Centre for Musculoskeletal Ageing Research, Division of Physiology, Pharmacology and Neuroscience, School of Life Sciences, University of Nottingham, Nottingham, UK; 4 University Hospitals of Derby and Burton NHS Foundation Trust, Derby, UK; 5 National Institute for Health Research Nottingham Biomedical Research Centre, Nottingham University Hospitals NHS Trust, Nottingham, UK; 6 University Hospitals of Leicester NHS Trust, Centre for Exercise and Rehabilitation Science, Glenfield Hospital, Leicester, UK; 7 Centre for Respiratory Research, Translational Medical Sciences, School of Medicine, University of Nottingham, City Hospital, Nottingham, UK; 8 Joint first authorship

## Abstract

**Background:**

Chronic obstructive pulmonary disease (COPD) patients exhibit lower peak oxygen uptake (*V*′_O_2___peak_), altered muscle metabolism and impaired exercise tolerance compared with age-matched controls. Whether these traits reflect muscle-level deconditioning (impacted by ventilatory constraints) and/or dysfunction in mitochondrial ATP production capacity is debated. By studying aerobic exercise training (AET) at a matched relative intensity and subsequent exercise withdrawal period we aimed to elucidate the whole-body and muscle mitochondrial responsiveness of healthy young (HY), healthy older (HO) and COPD volunteers to whole-body exercise.

**Methods:**

HY (n=10), HO (n=10) and COPD (n=20) volunteers were studied before and after 8 weeks of AET (65% *V*′_O_2___peak_) and after 4 weeks of exercise withdrawal. *V*′_O_2___peak_, muscle maximal mitochondrial ATP production rate (MAPR), mitochondrial content, mitochondrial DNA (mtDNA) copy number and abundance of 59 targeted fuel metabolism mRNAs were determined at all time-points.

**Results:**

Muscle MAPR (normalised for mitochondrial content) was not different for any substrate combination in HO, HY and COPD at baseline, but mtDNA copy number relative to a nuclear-encoded housekeeping gene (mean±sd) was greater in HY (804±67) than in HO (631±69; p=0.041). AET increased *V*′_O_2___peak_ in HO (17%; p=0.002) and HY (21%; p<0.001), but not COPD (p=0.603). Muscle MAPR for palmitate increased with training in HO (57%; p=0.041) and HY (56%; p=0.003), and decreased with exercise withdrawal in HO (−45%; p=0.036) and HY (−30%; p=0.016), but was unchanged in COPD (p=0.594). mtDNA copy number increased with AET in HY (66%; p=0.001), but not HO (p=0.081) or COPD (p=0.132). The observed changes in muscle mRNA abundance were similar in all groups after AET and exercise withdrawal.

**Conclusions:**

Intrinsic mitochondrial function was not impaired by ageing or COPD in the untrained state. Whole-body and muscle mitochondrial responses to AET were robust in HY, evident in HO, but deficient in COPD. All groups showed robust muscle mRNA responses. Higher relative exercise intensities during whole-body training may be needed to maximise whole-body and muscle mitochondrial adaptation in COPD.

## Abstract 2

**Muscle mitochondrial function is not impaired in age or COPD. Whole-body and mitochondrial exercise training adaptations are robust in young, evident in older and deficient in COPD. COPD adaptation may require high-intensity exercise training programmes.** https://bit.ly/3oe0k0S

## Introduction

Compared with age-matched healthy volunteers, patients with chronic obstructive pulmonary disease (COPD) exhibit lower whole-body maximal oxygen consumption [1–3] and abnormal skeletal muscle metabolic characteristics. Altered muscle fibre composition [4, 5], lower fibre cross-sectional area [6] and reduced capillarity [7] are apparent in COPD. At a mitochondrial level, lower ATP production capacity (reflected by lower maximal activities of mitochondrial enzymes [1]), altered mitochondrial efficiency [8], and both lower mitochondrial DNA (mtDNA) copy number and greater prevalence of mtDNA deletions [9] have been described in COPD. Collectively, these differences influence muscle metabolic responses to acute exercise, *e.g.* greater non-mitochondrial ATP production and adenine nucleotide loss [10], accentuating peripheral muscle fatigue development and reduced exercise capacity in COPD.

It is debated to what extent differences in muscle energy metabolism in COPD reflect muscle-level deconditioning (associated with reduced mitochondrial volume and/or number) and/or a disease-specific COPD mitochondropathy (characterised by impaired intrinsic capacity of mitochondrial units for respiration or ATP production) [8, 9, 11–15]. Similarly, chronological ageing has been linked with lower muscle mitochondrial protein content [16] and lower mitochondrial intrinsic capacity [17, 18] such that the aetiology of mitochondrial dysfunction in COPD could be age and/or disease related.

Aerobic exercise training (AET) robustly increases several markers of muscle mitochondrial content and function in young [19] and older [16] volunteers, but whether similar mitochondrial responses to AET occur in patients with COPD is uncertain, not least because ventilatory limitation in COPD can prevent skeletal muscle from being adequately challenged, thereby reducing training adaptation [20, 21].

Importantly, as far as we are aware, limited data are available depicting the impact of AET on markers of mitochondrial abundance and highly sensitive, direct measures of mitochondrial ATP production capacity in relation to whole-body cardiorespiratory adaptation in healthy, young and older volunteers and patients with COPD training concurrently. Similarly, these responses on return to habitual physical activity following training have not been depicted. Such data would provide insight regarding the aetiology of mitochondrial decline with age and COPD, and the mechanisms of exercise intervention in countering age- and COPD-related muscle decline and exercise intolerance. Such insight would also be beneficial to appreciate the utility of the mitochondrion as a therapeutic target.

We therefore aimed to determine the impact of matched relative intensity AET over an 8-week aerobic training regimen at 65% peak oxygen uptake (*V*′_O_2___peak_) and again 4 weeks following exercise withdrawal in healthy young and older volunteers and patients with COPD. Outcome measures include whole-body cardiorespiratory responses, markers of muscle mitochondrial content, maximal muscle mitochondrial ATP production rates (MAPRs) and the expression of mRNAs intimately linked to muscle fuel utilisation. We hypothesised that the response of MAPR and *V*′_O_2___peak_ to exercise intervention and withdrawal would differ between young and older healthy groups and between older healthy and COPD groups due to the impact of advanced age and disease pathology on muscle function and the potential impact of ventilatory limitation to exercise intensity in COPD.

## Methods

Additional detailed information for all methods is provided in the supplementary material.

### Subjects

Three groups of sedentary volunteers were recruited: patients with COPD (and significant exercise limitation due to breathlessness; Medical Research Council grade ≥3) aged 60–80 years (COPD), and age-matched healthy older (HO) and healthy young (HY) subjects aged 18–35 years (inclusion criteria are provided in supplementary table S1).

### Study design

A prospective, non-randomised interventional cohort study was performed. Anthropometry, pulmonary function and quadriceps strength were assessed at baseline and two symptom-limited incremental (the first being a familiarisation) and one submaximal cycling cardiopulmonary exercise test (CPET) performed. Seven-day physical activity levels were assessed (accelerometry) before a fasted state resting quadriceps muscle biopsy was obtained [22]. Participants commenced 8 weeks fully supervised cycling AET (three sessions of 30 min per week) at a workload corresponding to 65% *V*′_O_2___peak_. This intensity was chosen to elicit increases in muscle lipid and carbohydrate flux [23, 24]. Training intensity was reset at week 4 if workload at *V*′_O_2___peak_ had increased. After 8 weeks of training, participants were instructed to resume their pre-training habitual physical activity levels and were followed-up after 4 weeks (week 12). CPETs were repeated at weeks 4, 8 and 12. Further resting muscle biopsies were performed after 8 weeks (24 h after preceding training session) and 4 weeks post-exercise withdrawal (week 12). The study design is outlined in supplementary figure S1.

Ethical approval was granted by the NHS National Research Ethics Service and the trial was registered at the ISRCTN registry with identifier ISRCTN10906292. All participants provided written informed consent.

### Muscle mitochondrial preparation

Muscle samples were immediately subjected to a homogenisation, centrifugation and resuspension protocol, and then kept on ice immediately prior to quantification of MAPRs.

### Maximal MAPR (intrinsic mitochondrial function)

MAPR was assessed utilising the bioluminometric method described by Wibom *et al.* [25]. Briefly, diluted mitochondrial suspension, ADP and an ATP monitoring reagent (firefly luciferase) were combined with the following substrate combinations: glutamate and succinate; glutamate and malate; pyruvate and malate; palmitoyl-l-carnitine and malate (palmitate); succinate; and double-distilled water. All values were normalised to muscle citrate synthase activity.

### Citrate synthase activity

Muscle citrate synthase activity, a robust surrogate marker of mitochondrial content [26], was determined spectrophotometrically using the isolated mitochondrial suspension [27].

### Relative mtDNA copy number

The abundance of mitochondrial-encoded (mtDNA) NADH:ubiquinone oxidoreductase core subunit 1 was established using real-time PCR and expressed relative to genomically encoded (nuclear DNA) hydroxymethylbilane synthase as previously described [28].

### Targeted muscle mRNA expression

mRNA was extracted from snap-frozen muscle as previously described [29] and quantitative real-time PCR performed. The abundance of 59 transcripts involved in muscle fuel metabolism was assessed (supplementary material and supplementary table S2) and Ingenuity Pathway Analysis (IPA) (Qiagen, Hilden, Germany) was used to interrogate those differentially regulated.

### Statistical analysis

Data are presented as mean with standard deviation (baseline) or standard error of the mean (change over time). Assumptions of normality were assessed using normal probability plots and Shapiro–Wilk testing. Between-group mean differences at baseline were assessed using one-way ANOVA and within-group changes across time were tested using one-way repeated measures ANOVA with *post hoc* Fisher's least significant difference test using IBM SPSS Statistics for Windows version 21 (IBM, Armonk, NY, USA). A calculation of statistical power can be found in the supplementary material. For clarity, COPD *versus* HY comparisons are not reported due to lack of relevance to study aims.

## Results

### Baseline

Baseline subject characteristics are reported in table 1. Body mass index and fat-free mass index were not statistically different between groups at baseline. While all groups were non-active, the mean step count in COPD was lower than in HO (p=0.046) and below the sedentary lifestyle index for adults of 5000 steps per day [31].

**TABLE 1:** Baseline subject characteristics for the healthy older (HO), healthy young (HY) and chronic obstructive pulmonary disease (COPD) groups

	**HO (n=10)**	**HY (n=10)**	**COPD (n=20)**	**p-value three-way ANOVA**	**p-value HO *versus* HY**	**p-value HO *versus* COPD**
**Age, years**	70.7±5.1	28.0±5.1	70.2±5.9	<0.001	<0.001	0.834
**Female, n** **^#^**	5	6	14	0.556		
**FEV_1_, % pred**	98.3±9.2	112.6±20.6	55.6±16.2	<0.001	0.054	<0.001
**FEV_1_/FVC, %**	74.5±4.4	81.6±9.3	44.6±12.1	<0.001	0.126	<0.001
**RV, % pred**	98.4±19.2	107.9±48.9	157.3±44.9	0.001	0.603	0.001
**TLC, % pred**	104.3±11.9	107.6±15.0	121.3±19.1	0.010	0.667	0.007
**RV/TLC, %**	38.4±4.2	26.8±9.7	53.5±9.4	<0.001	0.005	<0.001
***T*_LCO_, % pred**	98.3±12.7	95.6±13.9	61.5±18.7	<0.001	0.714	<0.001
**Smoking history, n** **^#^**				<0.001		
Current smoker	0	0	0			
Never-smoker	6	10	0			
Ex-smoker	4	0	20			
**Smoking, pack-years** **^¶^**	18.3±21.5		38.5±15.4	0.036		0.036
**BMI, kg·m^−2^**	28.5±3.3	26.0±7.6	29.0±6.4	0.430		
**FFMI, kg·m^−2^**	18.1±1.5	16.5±2.8	17.2±2.7	0.379		
**QMVC isometric strength, Nm**	129.7±36.8	162.8±72.5	98.6±36.1	0.021	0.573	0.058
**Step count, 8-h average per day**	6007±2088 (n=9)	6180±3449 (n=9)	4012±1864 (n=19)	0.040	0.879	0.046

Data are presented as mean±sd, unless otherwise stated; percentage predicted values are calculated from normal values [30]. FEV_1_: forced expiratory volume in 1 s; FVC: forced vital capacity; RV: residual volume; TLC: total lung capacity; *T*_LCO_: transfer factor for the lung of carbon monoxide; BMI: body mass index; FFMI: fat-free mass index; QMVC: quadriceps maximum voluntary contraction. ^#^: differences in distribution between groups tested with the Pearson Chi-squared test; ^¶^: pack-year average for ex-smokers only.

Muscle citrate synthase maximal activity (mitochondrial content), MAPR (intrinsic mitochondrial function) and mtDNA copy number at baseline are shown in table 2. There was no statistical difference in citrate synthase activity between groups (p=0.070). Muscle mtDNA copy number was less in HO than in HY (p=0.041) (table 2). MAPR was not statistically different in HO compared with HY or COPD at baseline for any of the substrate combinations used (all p>0.05) (table 2).

**TABLE 2:** Baseline measures of quadriceps mitochondrial content and function for the healthy older (HO), healthy young (HY) and chronic obstructive pulmonary disease (COPD) groups

	**HO (n=10)**	**HY (n=10)**	**COPD (n=20)**	**p-value three-way ANOVA**	**p-value HO *versus* HY**	**p-value HO *versus* COPD**
**Citrate synthase activity, mmol acetyl-CoA·min^−1^·L mitochondrial suspension^−1^**	0.512±0.132	0.520±0.146	0.397±0.172	0.070		
**MAPR (glutamate and succinate), μmol ATP·mmol^−1^ acetyl-CoA·min^−1^**	1235±193	1249±142	1148±147	0.884		
**MAPR (glutamate and malate), μmol ATP·mmol^−1^ acetyl-CoA·min^−1^**	975±340	963±355	806±277	0.269		
**MAPR (pyruvate and malate), μmol ATP·mmol^−1^ acetyl-CoA·min^−1^**	658±245	570±161	477±195	0.073		
**MAPR (palmitate** **), μmol ATP·mmol^−1^ acetyl-CoA·min^−1^**	488±169	447±86	429±169	0.616		
**MAPR (succinate), μmol ATP·mmol^−1^ acetyl-CoA·min^−1^**	149±35	118±48	131±76	0.521		
**Relative mitochondrial DNA copy number^#^**	631±69	804±67	525±35	<0.001	0.041	0.140

Data are presented as mean±sd, unless otherwise stated. MAPR: maximal mitochondrial ATP production rate (normalised to citrate synthase activity for various mitochondrial substrates). ^#^: relative to nuclear DNA copy number.

During incremental exercise testing at baseline, HY reached a higher workload (p=0.001) and *V*′_O_2___peak_ (p<0.001) compared with HO, whereas *V*′_O_2___peak_ (p=0.026) and peak work rate (p=0.003) were less in COPD relative to HO (supplementary table S3). Measures coinciding with *V*′_O_2___peak_ showed that compared with HO, patients with COPD reached a lower respiratory exchange ratio (RER) (p<0.001), minute ventilation (*V*′_E_) (p=0.020) and heart rate (p=0.071), but utilised a significantly greater proportion of their predicted maximum voluntary ventilation (p<0.001) (supplementary table S3). Responses to submaximal steady-state exercise are shown in supplementary table S4.

### Influence of aerobic training and subsequent exercise withdrawal

**FIGURE 1:**
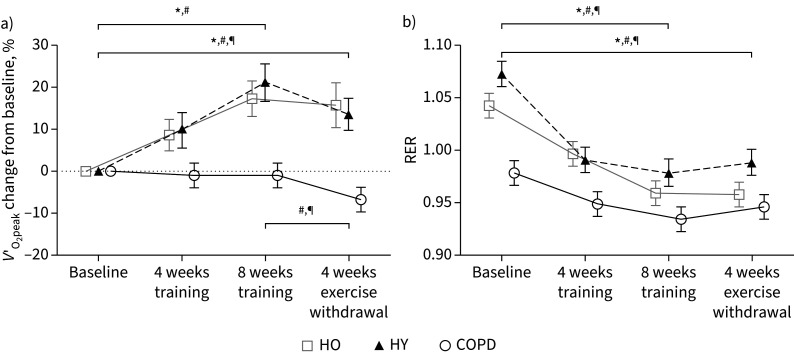
a) Within-group change in peak oxygen uptake (*V*′_O_2___peak_) normalised for lean body mass expressed as percentage change from baseline after 4 and 8 weeks of training and after 4 weeks of exercise withdrawal (where subjects returned to habitual physical activity levels) for healthy older (HO), healthy young (HY) and chronic obstructive pulmonary disease (COPD) groups. b) Respiratory exchange ratio (RER) during steady-state submaximal exercise at the same time-points as in (a). Data are presented as mean±sem. *: within-group change p<0.05 for HO; ^#^: within-group change p<0.05 for HY; ^¶^: within-group change p<0.05 for COPD.

Habitual physical activity levels in the first (week 9) and fourth (week 12) weeks of exercise withdrawal did not differ from baseline in any group (HO p=0.961, HY p=0.765 and COPD p=0.686) (supplementary table S5). HO and HY increased *V*′_O_2___peak_ by 18% (p=0.002) and 21% (p<0.001), respectively, following 8 weeks of training (figure 1a), but there was no change in *V*′_O_2___peak_ in COPD (p=0.603). Following 4 weeks of exercise withdrawal, *V*′_O_2___peak_ remained significantly greater than baseline in HO and HY (p=0.017 and p=0.004, respectively), while *V*′_O_2___peak_ was lower than baseline (p=0.026) and week 8 of exercise training (p=0.033) in COPD.

RER during steady-state submaximal exercise (65% of *V*′_O_2___peak_ at baseline) was reduced in all groups after 8 weeks of training (within-group change HO p<0.001, HY p<0.001 and COPD p=0.003) (figure 1b), and remained less than baseline after 4 weeks of exercise withdrawal (HO p<0.001, HY p<0.001 and COPD p=0.013). Heart rate during steady-state submaximal exercise decreased in all groups in the trained state (HO p=0.002, HY p<0.001 and COPD p=0.003) and *V*′_E_ was reduced in HO and HY (both p<0.05) as shown in supplementary figure S2.

**FIGURE 2:**
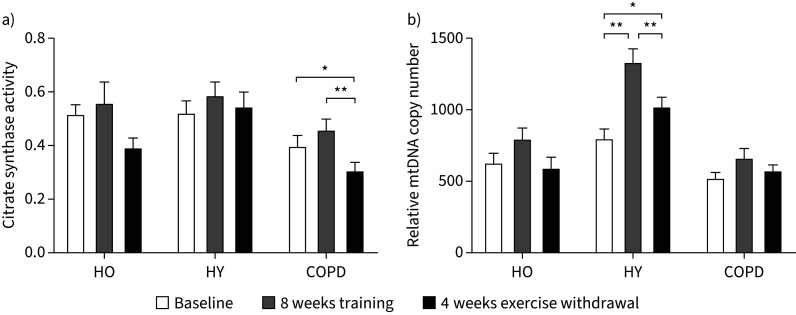
a) Muscle citrate synthase activity (mmol acetyl-CoA**·**min^−1^**·**L mitochondrial suspension^−1^) and b) mitochondrial DNA (mtDNA) copy number (relative to nuclear DNA copy number) at baseline, after 8 weeks of training and after 4 weeks of exercise withdrawal (where subjects returned to habitual physical activity levels) for healthy older (HO), healthy young (HY) and chronic obstructive pulmonary disease (COPD) groups. Data are presented as mean±sem. *: p<0.05; **: p<0.01.

There was no statistical increase in muscle citrate synthase activity in any group with training (HO p=0.120, HY p=0.682 and COPD p=0.133), but following 4 weeks of exercise withdrawal citrate synthase activity in COPD was less than baseline (p=0.015) and 8 weeks of training (p<0.001) (figure 2a). mtDNA copy number was statistically unchanged from baseline by AET and the return to habitual physical activity in HO (p=0.120) and COPD (p=0.132) (figure 2b). By contrast, there was a robust increase in mtDNA copy number with AET in HY (p<0.001), which declined on return to habitual physical activity (p<0.001), although remaining greater than baseline (p=0.024) (figure 2b).

**FIGURE 3:**
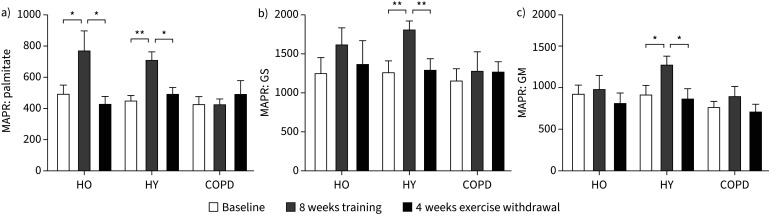
Muscle intrinsic mitochondrial function assessed by determining maximal mitochondrial ATP production rate (MAPR) (μmol ATP·mmol^−1^ acetyl-CoA·min^−1^) at baseline, after 8 weeks of training and after 4 weeks of exercise withdrawal (where subjects returned to habitual physical activity levels) for healthy older (HO), healthy young (HY) and chronic obstructive pulmonary disease (COPD) groups. a) Palmitate, b) glutamate and succinate (GS), and c) glutamate and malate (GM). Data are presented as mean±sem. *: p<0.05; **: p<0.01.

Exercise training intervention increased MAPR from baseline in HY for three substrate combinations (palmitate p=0.003; glutamate and succinate p=0.008; and glutamate and malate p=0.011) (figure 3), and for palmitate in HO (p=0.041) (figure 3a), but had no robust impact with any substrate combination in COPD (all p>0.05). Withdrawal of AET resulted in the return of MAPR to baseline rates in HY (palmitate p=0.016; glutamate and succinate p<0.001; and glutamate and malate p=0.003) and HO (palmitate p=0.036), but MAPR was unchanged from baseline in COPD (all substrates p>0.05) (figure 3). There was no change in MAPR from baseline for the mitochondrial substrates pyruvate and malate or succinate in any group of volunteers (all p>0.05; data not shown).

**FIGURE 4:**
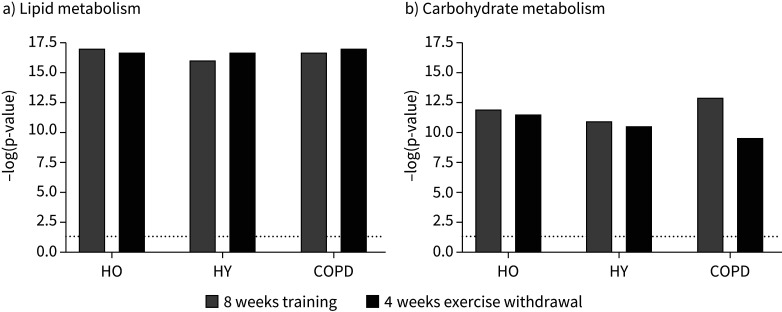
Muscle cellular functions identified by Ingenuity Pathway Analysis as being altered from baseline after 8 weeks of training and after 4 weeks of exercise withdrawal (where subjects returned to habitual physical activity levels) based on mRNA expression data generated using quantitative real-time PCR. a) Lipid metabolism and b) carbohydrate metabolism were significantly influenced after both training and exercise withdrawal. The *y*-axis displays −log(p-value) calculated by Fisher's exact test right-tailed. The dashed line denotes threshold of statistical significance for within-group change relative to baseline (−log(p-value)=1.3 is equivalent to p=0.05).

Based on changes in muscle mRNA expression from baseline, figure 4 illustrates that IPA identified both muscle lipid and carbohydrate metabolism to be altered following 8 weeks of training and 4 weeks of exercise withdrawal in HO, HY and COPD. The *y*-axis in figure 4 displays the −log of the p-value for each cellular function, which is a measurement of the likelihood that the association between a set of focus transcripts and a given function is due to random chance with the threshold of significance of the within-group change equivalent to p<0.05. Figure 4 illustrates that based on the collective change in mRNA abundance, the consistency of response of these two cellular functions to exercise training and 4 weeks of exercise withdrawal was very similar in HO, HY and COPD.

**FIGURE 5:**
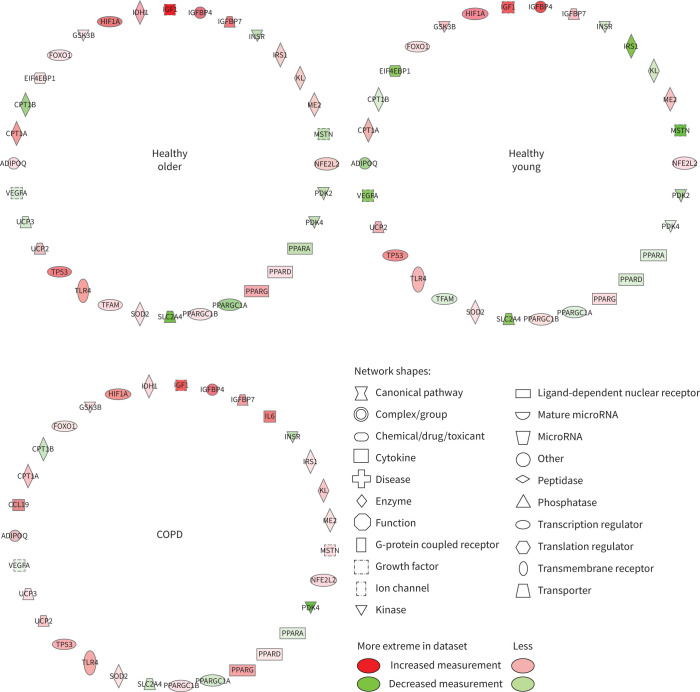
Differentially regulated muscle mRNAs associated with lipid metabolism following 8 weeks of training compared with baseline in healthy older, healthy young and chronic obstructive pulmonary disease (COPD) groups. Abbreviated gene names are defined in supplementary table S2.

**FIGURE 6:**
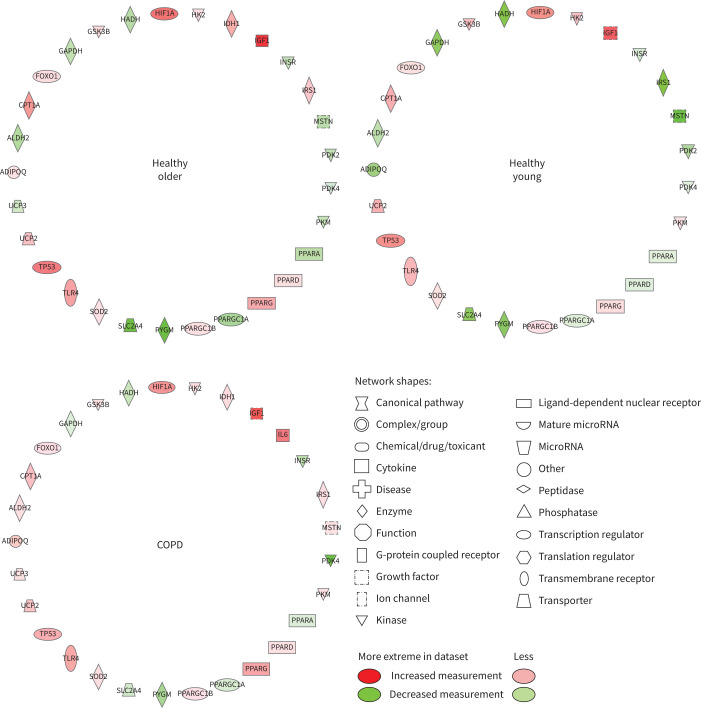
Differentially regulated muscle mRNAs associated with carbohydrate metabolism following 8 weeks of training compared with baseline in healthy older, healthy young and chronic obstructive pulmonary disease (COPD) groups. Abbreviated gene names are defined in supplementary table S2.

Figure 5 highlights the changes in mRNA abundance from baseline for individual genes identified by IPA to compromise the lipid metabolism function shown in figure 4 following 8 weeks of training. The number of mRNAs and the magnitude and direction of change of each were similar when comparing HO, HY and COPD. The same was true of individual mRNA expression changes from baseline after 4 weeks of exercise withdrawal for the lipid metabolism function (supplementary figure S3). Changes in mRNA abundance following 8 weeks of training (figure 6) and 4 weeks of exercise withdrawal (supplementary figure S4) for individual genes deemed to represent carbohydrate metabolism broadly followed the same magnitude and direction of change when comparing HO, HY and COPD.

## Discussion

The major novel findings of the present study are, first, that intrinsic mitochondrial function was not significantly different between the HO, HY and COPD groups at baseline, suggesting that mitochondrial function was not impaired in the basal state in patients with COPD or with healthy ageing. Second, changes in whole-body and muscle mitochondrial function in response to 8 weeks of endurance exercise training at the same relative intensity were robust in HY volunteers, less robust in HO volunteers and largely absent in COPD patients. These diminished responses could be a consequence of the lower absolute muscle loading experienced during training, particularly in COPD where the potential impact of ventilatory limitation to exercise training load exists, but the robust muscle transcriptional response to exercise intervention across all groups speaks against this and points to post-transcriptional regulation of muscle adaptation being different during exercise training. Finally, in keeping with the observation in HY volunteers [19], 4 weeks of exercise withdrawal returned intrinsic mitochondrial function to that seen in the baseline state in HY and HO volunteers.

It is debated to what extent changes in muscle energy metabolism in COPD reflect muscle-level deconditioning and/or a disease-specific COPD mitochondropathy [8, 9, 11–15]. Some data indicate that muscle mitochondrial respiration (corrected for total mitochondrial content) in patients with COPD is not impaired compared with older healthy volunteers [11, 13], as has been reported in other chronic disease states, *e.g.* type 2 diabetes [32]. Conversely, however, there is evidence that low muscle mitochondrial oxidative capacity in COPD cannot be explained by muscle-level deconditioning alone and is likely driven by the disease pathophysiology, including a shift away from complex I-driven respiration towards metabolically less-efficient complex II-driven respiration [8], greater mtDNA deletions, increased markers of abnormal fibre-specific respiration and suppressed mitochondrial proliferation (*e.g.* mtDNA copy number) [9, 12]. Given mitochondrial oxygen utilisation can be uncoupled from ATP production in pathophysiological states [33], a definitive conclusion as to whether a COPD-related mitochondropathy truly exists may be clouded by measurement of mitochondrial respiration as a surrogate of ATP generation [11, 13], a question addressed by the current study which utilised a sensitive measure of maximal MAPRs directly. These data, in combination with quantification of mitochondrial density (citrate synthase) and proliferation (mtDNA copy number), accord with the evidence that intrinsic mitochondrial function in patients is not impaired with ageing [16] or in the presence of COPD [11, 13].

Chronic AET increases several markers of muscle mitochondrial content and function in young [19] and older [16] volunteers, but limited information is available as to whether similar mitochondrial responses to AET occur in patients with COPD, not least because ventilatory limitation in COPD can prevent skeletal muscle from being adequately challenged during whole-body exercise, thereby reducing training adaptation [20]. High-intensity restricted muscle group training has been shown to restore mitochondrial function in patients with COPD to that observed in age-matched controls (albeit n=5), although it is unknown whether functional capacity also improved [13]. Picard *et al.* [11] also pointed to muscle deconditioning, rather than mitochondrial dysfunction, being the driver of lower maximal rates of mitochondrial respiration in patients with COPD. In the present study, exercise training of a large muscle mass was performed at an intensity high enough to increase mitochondrial lipid and carbohydrate oxidation, yet within the limits of tolerance for all groups. This exercise regimen produced robust changes in whole-body and muscle mitochondrial function and proliferation in HY volunteers, which were diminished in HO volunteers and absent in COPD patients. We acknowledge that muscle-level loading may have been truncated in the COPD group due to ventilatory limitation restricting whole-body exercise tolerance. Importantly, however, following exercise intervention the muscle transcriptional response of genes directly linked to muscle lipid and carbohydrate use was similar in patients with COPD compared with HO and HY volunteers. Furthermore, a decrease in steady-state RER during submaximal exercise across the course of 8 weeks of training was evident in all groups, demonstrating that chronic exercise adaptation in COPD was not limited by fuel mobilisation nor availability during exercise. It would appear rather that the training regimen employed was not sufficient to elicit adaptation in post-transcriptional regulation of mitochondrial biogenesis or mitochondrial function, particularly in COPD patients. This may result from ventilatory limitation restricting the magnitude of the muscle-level challenge; however, it has also been proposed that the plasticity of skeletal muscle adaptive responses to contractile activity is diminished with age and age-related disease, and this extends to mitochondrial proliferation and function, suggesting that a greater contractile stimulus is required to attain a similar phenotype adaptation [34]. Emerging sites for such post-transcriptional limitation include muscle translational efficiency (ribosome activity) and translational capacity (ribosome number) [35].

Remarkably, *V*′_O_2___peak_ and muscle citrate synthase activity in the COPD patients declined below the values recorded at baseline and following exercise training intervention, which was not observed in HY and HO volunteers. There is no obvious explanation for this observation given habitual physical activity levels during the first and fourth week of exercise withdrawal were not significantly different to that measured at baseline. However, it should be acknowledged that triaxial accelerometery, which was used to quantify step count in the current study, cannot quantify the intensity of the activities of daily living and therefore may perhaps explain the apparent deconditioning observed in the COPD group following 4 weeks of exercise withdrawal.

This study, through deliberate selection of sedentary healthy volunteers, better matched for habitual physical activity status of subjects compared with previous publications comparing COPD and health, and furthermore employed a tightly supervised exercise protocol. It is acknowledged that the sample size (although large for a study employing a demanding intervention and detailed metabolic and physiological measures) limits the generalisability of the findings to broader populations. The fat-free mass of patients with COPD in this study was normal, further limiting the generalisability of these findings to patients with low fat-free mass in whom differences in response to aerobic training (particularly at an mRNA level) have previously been observed [36]. Finally, a preferential loss of type I oxidative fibres has previously been observed in COPD, leaving a relatively large proportion of type II fibres compared with healthy controls [4, 5]. Distinct muscle fibre-type responses to exercise training would have added additional insight; however, due to limited tissue availability, it was not possible to characterise individual muscle fibre-type responses in this study. A further limitation of the current study is that it does not report muscle responses at a protein level downstream of changes in muscle mRNA abundance.

Our data align with the view that in COPD patients where maximal pulmonary ventilation is constrained, partitioned training strategies such as interval or restricted muscle group training aimed at maximising muscle-level training intensity are likely to stimulate greater muscle-level adaptation [21, 37] and thereby possibly patient benefit. Furthermore, the findings lend mechanistic support to current clinical rehabilitation guidelines recommending the prescription of high relative whole-body exercise intensities for people with COPD as low relative intensities may be insufficient to provoke robust whole-body and muscle-level adaptation [38]. It is also important to acknowledge the broader effects of pulmonary rehabilitation such as improvements in confidence, tolerance of breathlessness and mood that may impact on other clinical outcome measures that reflect patient benefit (*e.g.* field tests of exercise performance) regardless of relative exercise intensity. Nonetheless, enhancing adaptation at a muscle level has the potential to benefit patients and we suggest novel drug therapies targeting mitochondrial function should take account of the preservation of transcriptional responses to exercise training in the COPD group. Identifying the post-transcriptional locus of impairment is a critical research question to be addressed in the development of such therapies.

In conclusion, intrinsic mitochondrial function was not impaired with ageing or COPD. However, the magnitude of changes in whole-body responses to exercise, rates of mitochondrial ATP production and mitochondrial proliferation in response to chronic AET were variably blunted with age and further blunted in COPD, possibly as a result of ventilatory limitation. The similarity in muscle mRNA responses directly linked to fat and carbohydrate oxidation across HY, HO and COPD patient groups points to the plasticity of skeletal muscle to exercise-induced stress being diminished at the post-transcriptional level in COPD. Our findings are relevant to exercise prescription during pulmonary rehabilitation, suggesting that higher relative work intensities during whole-body aerobic training may be needed to stimulate adaptation at a muscle level in COPD and thereby maximise patient benefit.

## Supplementary material

10.1183/13993003.01507-2021.Supp1 **Please note:** supplementary material is not edited by the Editorial Office, and is uploaded as it has been supplied by the author.
Supplementary material ERJ-01507-2021.Supplement

## Shareable PDF

10.1183/13993003.01507-2021.Shareable1 This one-page PDF can be shared freely online.
Shareable PDF ERJ-01507-2021.Shareable
