# Supplementary material for: Whole-body and muscle responses to aerobic exercise training and withdrawal in ageing and COPD
Source: Eur Respir J. 2022 May 12;59(5):2101507. doi: 10.1183/13993003.01507-2021 (PMC9095946; doi:10.1183/13993003.01507-2021)
Supplement: Supplementary file 1 [file ERJ-01507-2021.Supplement.pdf]

Online Data Supplement

Whole-body & muscle responses to aerobic exercise training and withdrawal in ageing & COPD

Lorna E Latimer, Dumitru Constantin-Teodosiu, Bhavesh Popat, Despina Constantin, Linzy Houchen-Wolloff, Charlotte E Bolton, Michael C Steiner, Paul L Greenhaff

METHODS

Subjects

Participants for all groups were identified from existing research databases; patients with COPD were recruited from outpatient clinics; and the healthy volunteers were identified via local advertisement. Participants were not engaged in regular exercise training prior to enrollment (<150 min moderate intensity exercise / week and patients had not attended pulmonary rehabilitation in preceding 12 months). Inclusion and exclusion criteria are in Table S1.

Table S1. Participant inclusion / exclusion criteria.

	**Inclusion**	**Exclusion**
**Young Healthy Adults (HY)**	Age $\geq$18 & $\leq$35	
	Not engaged in regular exercise programme	Exceeding 150 min / week moderate intensity exercise
	Normal lung function: FEV_1 >80% predicted, FEV_1 / FVC $\geq$70%	Any respiratory diagnosis
**Healthy Older Adults (HO)**	Age $\geq$60 & $\leq$80	
	Not engaged in regular exercise programme	Exceeding 150 min / week moderate intensity exercise
	Normal lung function: FEV_1 >80% predicted, FEV_1 / FVC $\geq$70%	Any respiratory diagnosis

**COPD**	Age $\geq$60 & $\leq$80	
	Clinical diagnosis of COPD and obstructive spirometry: FEV_1 <80% predicted, FEV_1 / FVC <70%	
	Not engaged in regular exercise programme	Attended PR within last 12 months
	MRC grade $\geq$3	
	Clinically stable	Exacerbation within last 4 weeks
**All Groups**	Ability to give informed consent	Any medical condition associated with metabolic disturbance (e.g. type II diabetes), inflammation (e.g. rheumatoid arthritis, inflammatory bowel disease), impaired muscle function or one which affects the ability to perform exercise testing and training (e.g. cardiovascular disease, significant osteoarthritis)
		Receiving systemic corticosteroid medication
		Receiving anticoagulation therapy or condition causing impaired clotting / platelet dysfunction
		Current smoker (ex-smokers < 1 year were acceptable)

Study design

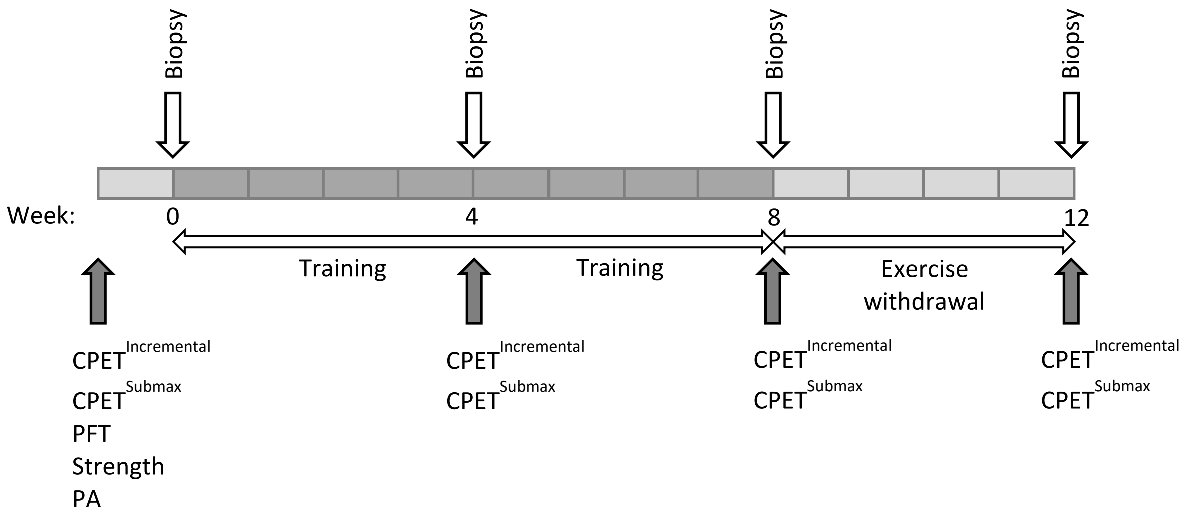

Figure S1. Study protocol followed by all volunteers. Biopsy, microbiopsy of *vastus lateralis*; $CPET^{Incremental}$, symptom-limited incremental cardiopulmonary exercise test; $CPET^{Submax}$, submaximal cardiopulmonary exercise test at workload corresponding to 65% of the workload at $\dot{V}O_2^{PEAK}$ in baseline $CPET^{Incremental}$; PFT, pulmonary function test; strength, quadriceps maximal voluntary contraction; PA, physical activity monitoring for 7 days. Training intensity was reset at week four if workload at $\dot{V}O_2^{PEAK}$ had increased.

Baseline assessments

Baseline assessments were performed over three visits. At visit 1 measures of anthropometry (height, body mass, body composition by DEXA), pulmonary function and quadriceps strength were performed as was a familiarisation symptom-limited incremental cycling cardiopulmonary exercise test (incremental CPET). At the second visit (minimum 48 hours after visit one) a further incremental CPET (to verify the preceding test) was performed

followed by a submaximal cycling exercise tests (separated by $\geq$30 minutes resting time). A minimum of 7 days later, at the third visit, a quadriceps muscle biopsy was performed (week 0). Habitual physical activity was assessed over seven days prior to intervention and repeated during the first and last weeks of the exercise withdrawal period.

Anthropometry, pulmonary function, quadriceps strength and physical activity monitoring

Measurements of pulmonary function including lung volumes by plethysmography [S1], body mass index (BMI), body composition (dual energy x-ray absorptiometry, DEXA; Lunar Prodigy, GE Healthcare, Buckinghamshire, United Kingdom), and quadriceps isometric strength at 90° knee and hip flexion (Cybex II Norm: CSMi, Stoughton, USA) were performed at baseline. Habitual physical activity was monitored using a triaxial accelerometer (SenseWear; BodyMedia, Pittsburgh, USA) worn during waking hours on 7 consecutive days before baseline, and during the first (week 9) and final (week 12) week of exercise withdrawal. A minimum of 8 hours of data per day was required to be included in the analysis. The mean step count of the initial 8 hours after waking was calculated for each individual.

Incremental CPET and a submaximal cycling exercise tests

Incremental CPET was performed in accordance with international guidelines [S2] on an electromagnetically braked cycle ergometer (Lode Corival, Groningen, Netherlands) with continuous electrocardiogram, oxygen saturation and blood pressure monitoring. After three min unloading pedalling, workload was increased progressively (ramp protocol) at a rate between 5 W/min and 20 W/min so as to elicit a test of 8 – 12 min duration. All subsequent incremental tests employed the same protocol as the baseline test. Breath by breath measurements of gas exchange ($\dot{V}O_2$, $\dot{V}CO_2$) and ventilation ($\dot{V}E$) were monitored using a metabolic cart (Ergocard Professional, Medisoft, Sorinnes, Belgium) and reported as fifteen

breath rolling averages. Predicted maximum voluntary ventilation (MVV) was calculated as FEV_1 x 37.5 (S3).

Sub-maximal testing was performed at 65% of the workload corresponding to the highest $\dot{V}O_2^{PEAK}$ achieved in either of the baseline tests and was not changed for subsequent tests. Continuous monitoring of cardiorespiratory variables was performed throughout the test and an average reported for the steady-state period. The steady-state period excluded the first three minutes at test workload and isotime measures were calculated for each individual, the duration of which was determined by the shortest test performed by that individual (normally the baseline test). The sub-maximal test was terminated by the investigators after 30 min. The intensity of the sub-maximal test corresponded to the initial training intensity and measures of heart rate, $\dot{V}E$, and respiratory exchange ratio (RER) indicate the physiological stress experienced during training.

Muscle sampling and processing

Muscle biopsy samples were obtained from the *vastus lateralis* muscle of the dominant leg at mid-thigh level using a needle micro-biopsy technique [S4]. Briefly, after the skin was sterilised with Betadine solution, local anaesthetic (lignocaine) was injected subcutaneously and to the depth of the fascia. A small incision (5 mm) was made in the skin and any subcutaneous adipose, through which a 12 g micro-biopsy needle was inserted (Bard Magnum, Arizona, USA). Four passes were performed, each harvesting ~20 mg of tissue. Approximately 40 mg of freshly isolated vastus lateralis muscle tissue was finely diced on a cooled glass plate, and weighed for mitochondrial function and content measurements. The remaining muscle tissue was immediately dissected free of visible adipose and connective

tissue, snap frozen and stored in liquid nitrogen for subsequent DNA and mRNA analyses. The biopsy site was dressed with a butterfly closure, waterproof sterile dressing and a compression bandage to apply light pressure in order to minimise the risk of bleeding or bruising. Subsequent biopsies were performed 2.5 cm from the preceding incision site.

Muscle Mitochondrial measurements

Each sample was homogenised on ice for 3 min in a buffer solution (pH 7.0, KCl 100mM, KH_2PO_4 50 mM, Tris 50 mM, $MgCl_2$ 5mM, EDTA 1 mM, ATP 1.8 mM) using a Teflon pestle homogeniser. The crude homogenate was then centrifuged at 650 g for 3 min at 4^oC, and the resultant supernatant was transferred to a test tube and centrifuged at 15,000 g for 3 min at 4^oC. The resulting pellet formed contained the mitochondria. Following this, the supernatant was removed and discarded before resuspension of the pellet in 300 µl of the original homogenisation buffer. This was then centrifuged at 15,000 g for 3 min at 4^oC. After removal of the supernatant, the pellet was re-suspended in a re-suspension solution (pH 7.0, human serum albumin 0.5 mg/ml, sucrose 240 mM, monopotassium phosphate 15 mM, magnesium acetate tetrahydrate 2mM, EDTA 0.5 mM). The differential centrifugation isolated mitochondrial suspension was then kept on ice immediately prior to measurement of mitochondrial ATP production rates (MAPR).

Mitochondrial ATP production rates (intrinsic mitochondrial function)

Following the method of Wibom et al [S5], 2.5 µl of diluted mitochondrial suspension was added to each well of a luminometer plate. The wells contained 200 µl ATP monitoring reagent (Firefly Luciferase, 357 µM D-luciferine, 14.3 µM L-luciferine, 15 mM BSA, 1 µM Sodium Pyrophosphate Decahydrate, 187.55 mM Sucrose, 18.8 mM monopotassium phosphate, 2.5 mM magnesium acetate tetrahydrate, 677 µM EDTA (K Salt) pH 7.0), 12.5 µl

of 12 mM ADP and 35 µl of the following substrate combinations in duplicate: Glutamate (16.4 mM) and Succinate (15 mM); Glutamate (32.75 mM) and Malate (22 mM); Pyruvate (50 mM) and Malate (22 mM); Palmitoyl-l-carnitine (5 µM) and Malate (1.5 mM); Succinate (2.5 mM); and double-distilled water (ddH_2O). A blank that contained all the above components except the substrate was run in parallel for each sample. Luminescence was continuously recorded for 10 min (BMG LABTECH, Ortenberg, Germany) and an injection of 150 pM ATP standard took place after 4.6 min. The change in luminescence elicited by the ATP standard was used to calculate MAPR. Mitochondrial suspensions were frozen at -80°C for subsequent determination of citrate synthase activity (see below). MAPR values were corrected for maximal citrate synthase activity (routinely used as an index of mitochondrial content / mass) to provide measures of intrinsic mitochondrial function normalised to the mitochondrial content of the suspension.

Citrate synthase activity (mitochondrial content)

Muscle citrate synthase (CS) maximal activity was determined at 37°C on the isolated mitochondrial solution using a kinetic spectrophotometric method to follow the change in absorbance of a 5,5'-dithiobis (2-nitrobenzoic acid)(DNTB) buffered solution as previously described (E6). Briefly, 15 µl mitochondrial suspension was added to 185 µl homogenisation buffer (95% extraction buffer containing 1% triton) and was homogenised using a glass pestle at 200 rpm for 2 min (5). The homogenate was centrifuged at 24,000 g (Eppendorf, Hamburg, Germany) before CS was determined spectrophotometrically in the supernatant [S5]. Acetyl-CoA is formed at a rate determined by the quantity of CS protein present.

Relative mitochondrial DNA copy number Genomic DNA (nDNA) and mitochondrial DNA (mtDNA) were extracted from skeletal muscle Qiagen using a QIAamp® DNA Mini kit

according to the manufacturer's instructions. Briefly, the procedure involved initial tissue lysis in a buffer containing proteinase K, incubation for 3 hrs at 56°C to digest the myofibril proteins followed by the spinning of the lysates on silica-membrane-based nucleic acid purification columns and elution of the mtDNA and nDNA with appropriate buffers. Before the addition of buffer AL (Qiagen), 4 µL of free DNase activity RNase A stock solution 7000 U/mL was added to each sample lysate. DNA quality and quantity was assessed by measurement of light at 260, 280 and 230 nm (Nanodrop One Spectrophotometer, Thermoscientific, Waltham, MA, USA). The expression level of hydroxymethylbilane synthase (HMBS; nDNA) and encoded NADH:ubiquinone oxidoreductase core subunit 1 (ND1; mtDNA) was used to evaluate the relative abundance of nuclear and mitochondrial DNA and was quantified using TaqMan probe real-time PCR. The TaqMan probe design for the detection of nDNA levels was based on interrogation of the intron sequence spanning between exons 3-4 of the genomic hydroxymethylbilane synthase (HMBS) gene to avoid any mRNA amplification, if present. The probe design for detection of mtDNA levels was based on interrogation of a stable fragment of the mtDNA loop, namely the mitochondrially encoded NADH:ubiquinone oxidoreductase core subunit 1 (ND1). The $2^{-\Delta Ct}$ formula, where $\Delta = Ct_{ND1}\text{-}Ct_{HMBS}$, was used to express the relative number of mtDNA copies.

Muscle mRNA expression linked to fuel metabolism

RNA was extracted from ~30 mg snap-frozen muscle as previously described [S7]. First strand cDNA was synthesised from 1 µg of total RNA, using Superscript III reverse transcriptase (Invitrogen Ltd, Paisley, UK) and random primers (Promega, Southampton, UK) and stored at -80°C until analysis. TaqMan low density arrays were performed using an ABI PRISM 7900HT sequence detection system, and data analysed using SDS 2.1 software (Applied Biosystems,

USA). Data were further analysed using RQ Manager software (Applied Biosystems, USA), where the threshold level was normalised across all plates before Ct values were calculated for each gene target and sample. Relative quantification of mRNAs of interest was measured using the $2^{-\Delta\Delta Ct}$ method with hydroxymethylbilane synthase (HMBS) as the endogenous control as it was unaffected by exercise intervention (data not shown). A total of 59 transcripts known to be involved in muscle carbohydrate and lipid metabolism were targeted for analysis in the present study (Table S1). Target selection was led by published data involving high-throughput and targeted RT-PCR approaches from our research group which identified muscle transcripts responsive to exercise intervention [S8], insulin resistance [S9] and changes in fuel metabolism with nutritional [S10] and pharmacological intervention [S11]. Additionally unpublished muscle transcript data from our group from research involving limb immobilisation in healthy volunteers was accessed. To associate altered biological functions to the targeted probe sets, Ct values were uploaded to Ingenuity Pathway Analysis (IPA) software (QIAGEN, Hilden, Germany) for pathway analysis of gene expression data. The overall outcome of IPA (e.g. cellular function) is predicted by calculating a regulation Z-score and an overlap p-value, which are based on: 1, the number of regulated target genes' function; 2, the magnitude of expression change; 3, the direction of expression change; and 4, their concordance with the IPA database, which is constructed from an extensive curated literature database. The overlap p-value was calculated by IPA to identify significantly enriched function pathways from the submitted list of significantly changed genes. These p-values were generated from the right-tailed Fisher's Exact Test, and a significance threshold of $p<0.05$ was used to assess the statistical significance of the function pathways. In order to control for any enrichment of false positive results when undertaking multiple comparisons (type II errors) IPA utilises Bonferroni's corrected p-value set at $p< 0.05$.

Table S2. mRNA transcripts with functions relating to muscle fuel metabolism that were quantified in this analysis.

Abbreviation	Gene name	ABI ID
**ALDH2**	Aldehyde dehydrogenase 2 family (mitochondrial)	Hs01007998_m1
**G6PD**	Glucose-6-phosphate dehydrogenase	Hs00166169_m1
**GAPDH**	glyceraldehyde-3-phosphate dehydrogenase	Hs99999905_m1
**GLUD1**	glutamate dehydrogenase 1	Hs03989560_s1
**GYS1**	glycogen synthase 1 (muscle)	Hs00157863_m1
**HADH**	[illegible]-hydroxyacyl-CoA dehydrogenase	Hs00193428_m1
**HK1**	hexokinase 1	Hs00175976_m1
**HK2**	hexokinase 2	Hs00606086_m1
**IDH1**	Isocitrate dehydrogenase 1	Hs00271858_m1
**PDP2**	pyruvate dehyrogenase phosphatase catalytic subunit 2	Hs00380020_m1
**PFKM**	phosphofructokinase, muscle	Hs00175997_m1
**PGM1**	phosphoglucomutase 1	Hs01071897_m1
**PKM**	pyruvate kinase, muscle	Hs00987255_m1
**PYGM**	phosphorylase, glycogen, muscle	Hs00989942_m1
**ACOT1**	acyl-CoA thioesterase 1	Hs04195130_s1
**ADIPOQ**	adiponectin	Hs00605917_m1
**APOA1**	Apolipoprotein A-I	Hs00163641_m1
**APOC1**	Apolipoprotein C	Hs00155790_m1
**CCL19**	chemokine (C-C motif) ligand 19	Hs00171149_m1

**CPT1A**	carnitine palmitoyltransferase, liver isoform	Hs00912671_m1
**CPT1B**	carnitine palmitoyltransferase, muscle isoform	Hs00189258_m1
**EIF4EBP1**	eukaryotic translation initiation factor 4E binding protein 1	Hs00607050_m1
**FAXDC2**	fatty acid hydroxylase domain containing 2	Hs00260753_m1
**FOXO1**	Forkhead box protein O1	Hs01054576_m1
**HSPA14**	heat shock 70kDa protein 14	Hs00212495_m1
**HSPD1**	heat shock 60kDa protein 1 (chaperonin)	Hs01036747_m1
**IDH1**	Isocitrate dehydrogenase 1	Hs00271858_m1
**IGFBP4**	insulin-like growth factor binding protein 4	Hs01057900_m1
**IGFBP7**	insulin-like growth factor binding protein 7	Hs00266026_m1
**IL6**	interleukin 6 (interferon, beta 2)	Hs00985639_m1
**KL**	klotho	Hs00183100_m1
**LOXHD1**	lipoxygenase homology domains 1	Hs00329848_m1
**LPPR2**	lipid phosphate phosphatase-related protein type 2	Hs01106565_m1
**ME2**	malic enzyme 2, NAD(+)-dependent, mitochondrial	Hs00929809_g1
**NFE2L2 (NRF2)**	nuclear factor, erythroid 2 like 2	Hs00975961_g1
**NRF1**	nuclear respiratory factor 1	Hs00192316_m1
**PLA2**	Phospholipase A2	Hs00179898_m1
**PLD4**	phospholipase D family, member 4	Hs00975488_m1

**PPARA**	peroxisome proliferator-activated receptor alpha	Hs00947536_m1
**PPARD**	peroxisome proliferator-activated receptor delta	Hs00987011_m1
**PPARG**	peroxisome proliferator-activated receptor gamma	Hs01115513_m1
**PPARGC1A**	peroxisome proliferator-activated receptor gamma, coactivator 1 alpha	Hs01016719_m1
**PPARGC1B**	peroxisome proliferator-activated receptor gamma, coactivator 1 beta	Hs00991677_m1
**SOD2**	superoxide dismutase, mitochondrial	Hs00167309_m1
**TFAM**	transcription factor A, mitochondrial	Hs00273372_s1
**TLR4**	toll-like receptor 4	Hs00152939_m1
**TP53 (p53)**	tumor protein p53	Hs01034249_m1
**UCP2**	uncoupling protein 2 in mitochondria	Hs01075227_m1
**UCP3**	uncoupling protein 3 in mitochondria	Hs01106052_m1
**VEGFA**	Vascular endothelial growth factor A	Hs00900055_m1
**GSK3B**	Glycogen synthase kinase-3 beta	Hs01047719_m1
**HIF1A**	Hypoxia-inducible factor 1-alpha	Hs00153153_m1
**IGF1**	insulin-like growth factor 1 (somatomedin C)	Hs01547656_m1
**INSR**	Insulin receptor	Hs00961554_m1
**IRS1**	Insulin receptor substrate 1	Hs00178563_m1
**MSTN**	myostatin	Hs00976237_m1
**PDK2**	pyruvate dehydrogenase kinase, isozyme 2	Hs00176865_m1

**PDK4**	pyruvate dehydrogenase kinase, isozyme 4	Hs01037712_m1
**SLC2A4 (GLUT4)**	glucose transporter	Hs00168966_m1

Exercise training intervention

Participant underwent supervised training on an electrically braked cycle ergometer (Lode Corival, Groningen, The Netherlands). Three supervised sessions of 30 min duration were performed per week. Individuals who were unable to compete 30 min continuously were permitted to rest (~5 min) before resuming the session over a total permissible duration of 60 min. Exercise at the prescribed intensity (workload corresponding to 65% $\dot{V}O_2^{PEAK}$) is known to increase muscle lactate accumulation, pyruvate dehydrogenase complex (PDC) activation and flux [S12], and rates of mitochondrial carbohydrate and lipid oxidation from both plasma and muscle sources [S13] well above the resting state, thereby providing a robust stimulus to muscle metabolic adaptation.

Statistical analysis

A power calculation performed on MAPR data (glutamate and succinate) using G-Power software (version 3.1.9.2, Dusseldorf University, Germany) for ANOVA one-way fixed effects given $\alpha = 0.05$, number of groups = 3, power = 0.9 and effect size = 0.6 using the data from Barany et al. [S14] recommended n=9 for the healthy control group, which we rounded up to n=10 for healthy volunteers. Given the inherently variable nature of physiological responses in patients with COPD, this number was increased to n=20 in the COPD group.

Approvals & clinical trial registration

The trail was approved by NHS National Research Ethics Service, West Midlands Committee – Coventry & Warwickshire (reference 13/WM/0075) and registered with the UK Clinical

Research Network (UK CRN) ID: 14080. This trial was registered with www.isrctn.com, reference 10906292.

RESULTS

Baseline

Fourteen HO and 15 HY volunteers consented to participate with 10 from each group completing all study measures. Twenty-seven patients with COPD consented, 20 of whom completed the training intervention and week eight assessments with one drop-out during the exercise withdrawal period leaving 19 COPD patients in the week 12 analysis.

Table S3. Cardiorespiratory measures at $\dot{V}O_2^{PEAK}$ in the baseline incremental test

	**Healthy Older (n=10)**	**Healthy Younger (n=10)**	**COPD (n=20)**
**$\dot{V}O_2^{PEAK}$**, *ml.kg lean mass^{-1} min^{-1}*	29.7 (4.0)	44.7 (5.6)**	24.0 (7.4)*
**Work load,** *W*	113.0 (26.4)	167.0 (43.6)**	71.1 (32.5)**
**RER**	1.20 (0.09)	1.23 (0.10)	1.05 (0.09)**
**$\dot{V}E$,** *L min^{-1}*	62.8 (16.4)	82.9 (25.5)*	44.9 (16.6)*
**$\dot{V}E$/MVV,** *%*	65.4 (15.9)	65.2 (17.0)	103.4 (23.8)**
**HR % Predicted Max,** *%*	94.4 (10.2)	97.0 (4.8)	85.8 (11.2)*

RER, respiratory exchange ratio; $\dot{V}E$, minute ventilation; MVV, predicted maximum voluntary ventilation; HR, heart rate predicted maximun = 220 – age (years). Values are mean (SD). *, $p < 0.05$ *vs* HO; **, $p < 0.05$ *vs* HO.

Table S4. Baseline physiological responses to steady-state sub-maximal cycling exercise at an intensity corresponding to 65% of the work load achieved at baseline $\dot{V}O_2^{PEAK}$.

	**Healthy Older (n=9)**	**Healthy Younger (n=10)**	**COPD (n=20)**
**Work load,** *W*	76 (18)	110 (26)**	46 (21)**
**RER**	1.04 (0.34)	1.07 (0.37)	0.98 (0.53)**
**$\dot{V}E$,** *$L\ min^{-1}$*	52.2 (11.4)	66.3 (16.9)*	35.7 (11.6)**
**$\dot{V}E$/MVV,** *%*	53.8 (11.9)	53.4 (15.9)	83.6 (18.8)**
**HR % Predicted Max,** *%*	86.8 (11.9)	92.6 (3.9)	79.6 (12.1)
Data represent the steady-state period of a cycling exercise test at an individually prescribed work rate (65% of the work rate that corresponded with baseline $\dot{V}O_2^{PEAK}$). RER, respiratory exchange ratio; $\dot{V}E$, minute ventilation; MVV, predicted maximum voluntary ventilation; HR, heart rate predicted maximun = 220 – age (years). Values are mean (SD). *, *p* < 0.05 *vs* HO; **, *p* < 0.01 *vs* HO.			

There were no significant within-group changes over time in physical activity assessed by daily step count (Table S5).

Table S5. Daily step count.

Steps in 8 hours	Baseline	Exercise Withdrawal Week 1	Exercise Withdrawal Week 4	*p* (within group)
Healthy Older	6007 (2088) (n=9)	3976 (579) (n=9)	6012 (2885) (n=9)	NS
Healthy Younger	6180 (3449) (n=9)	6384 (3217) (n=9)	5778 (3212) (n=7)	NS
COPD	4012 (1861) (n=19)	3983 (2077) (n=18)	3772 (2083) (n=18)	NS
Physical activity (daily step count) at baseline (pre-training) and in the 1st and 4th week of the exercise withdrawal period. Data are mean (SD).				

Heart rate during steady state exercise was reduced from baseline after eight weeks training and after four weeks exercise withdrawal in all groups (Fig. S2 A; all $p < 0.01$) and $\dot{V}E$ was reduced at the same time points in HO ($p < 0.01$) and HY ($p < 0.05$) but was unchanged in COPD (Fig. S2 B).

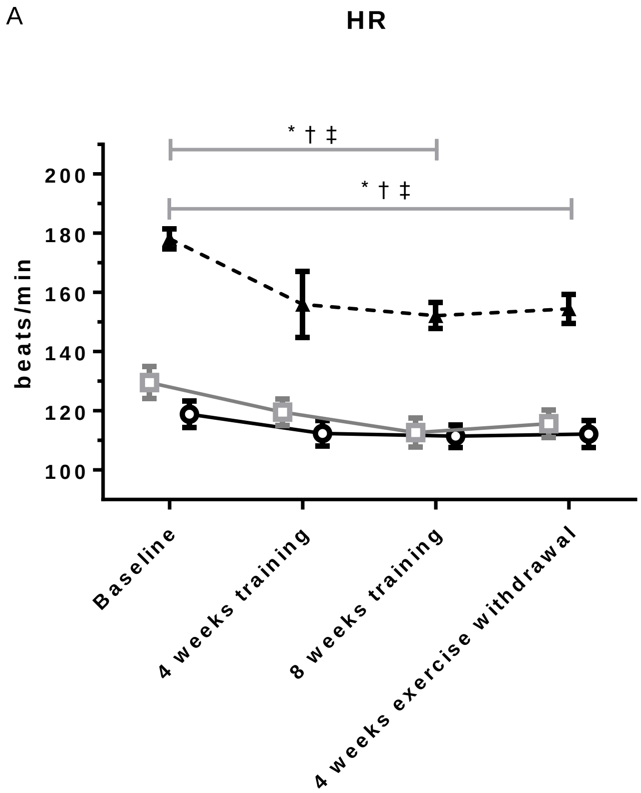

A
HR
* † ‡
* † ‡
beats/min
200
180
160
140
120
100
Baseline
4 weeks training
8 weeks training
4 weeks exercise withdrawal

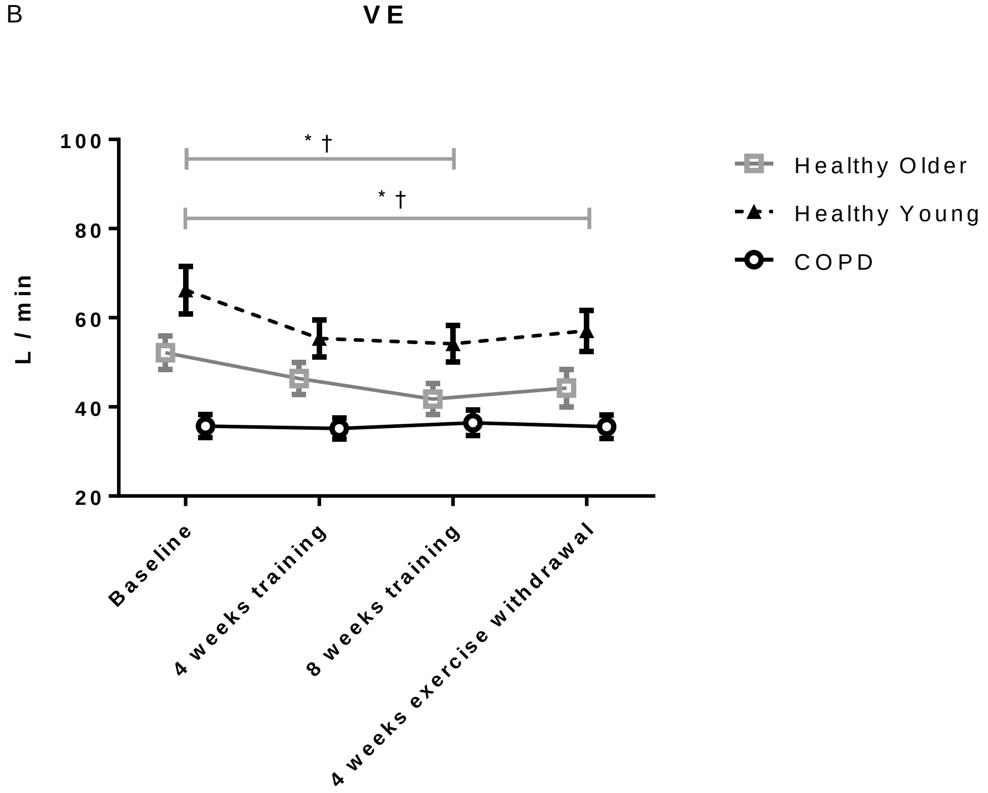

B
VE
* †
* †
L / min
100
80
60
40
20
Baseline
4 weeks training
8 weeks training
4 weeks exercise withdrawal
Healthy Older
Healthy Young
COPD

Figure S2. Physiological responses to steady-state sub-maximal exercise at a 65% of the work load achieved at $\dot{V}O_2^{PEAK}$ in the baseline test. A, Change in heart rate (HR) after 8 weeks training, and after 4 weeks exercise withdrawal where subjects returned to habitual physical activity levels. B, Respiratory exchange ratio (RER) at the same time points as above (A). Within group change $p < 0.05$ for: HO, *; HY, †; COPD, ‡. Values are mean (SEM).

Lipid Metabolism (After 4 Weeks Exercise Withdrawal)

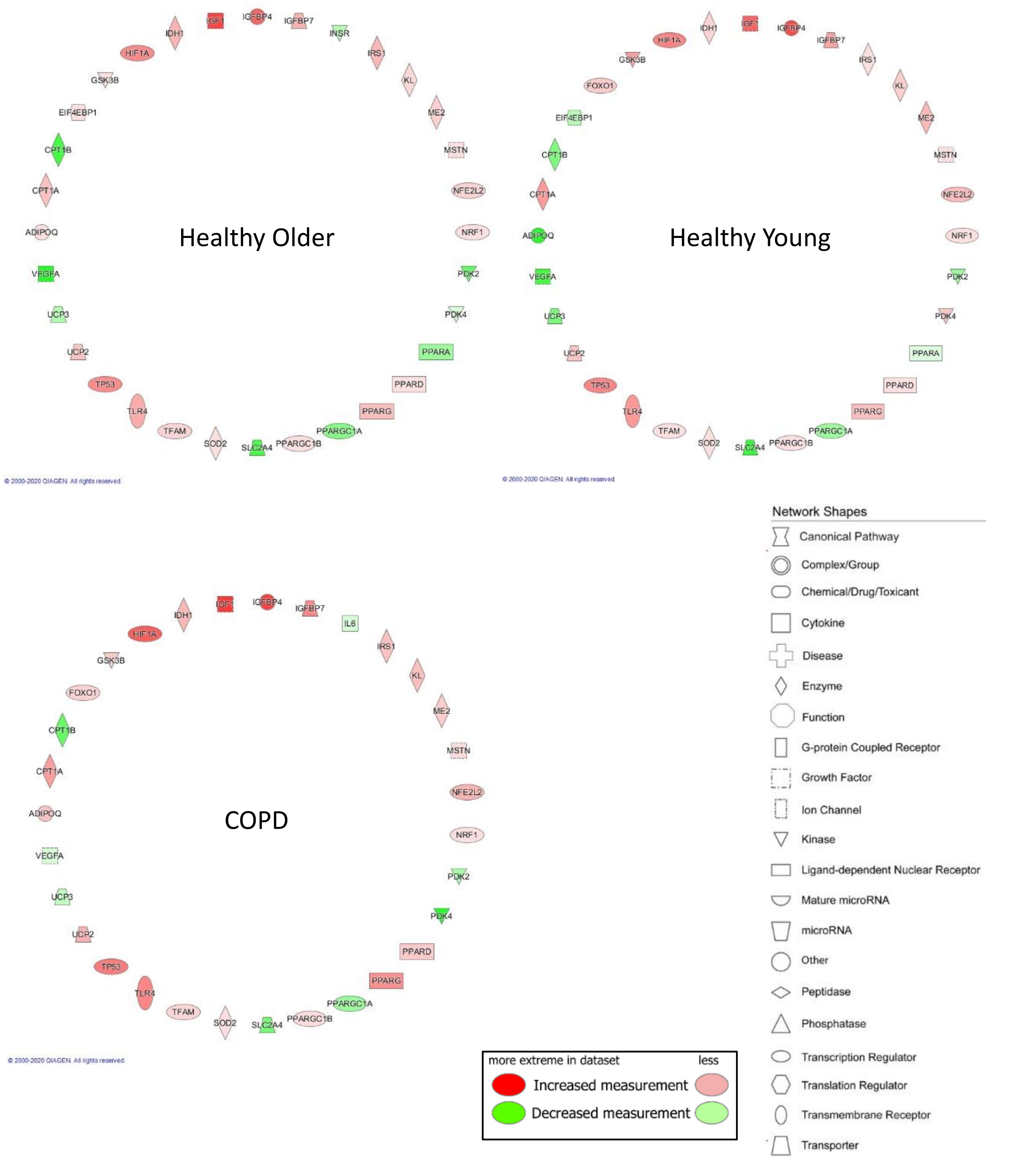

Figure S3. Differentially regulated muscle mRNAs associated with lipid metabolism following 4 weeks exercise withdrawal compared to baseline in healthy older, healthy young and COPD groups. Abbreviated gene names are defined in Table S2.

Carbohydrate Metabolism (After 4 Weeks Exercise Withdrawal)

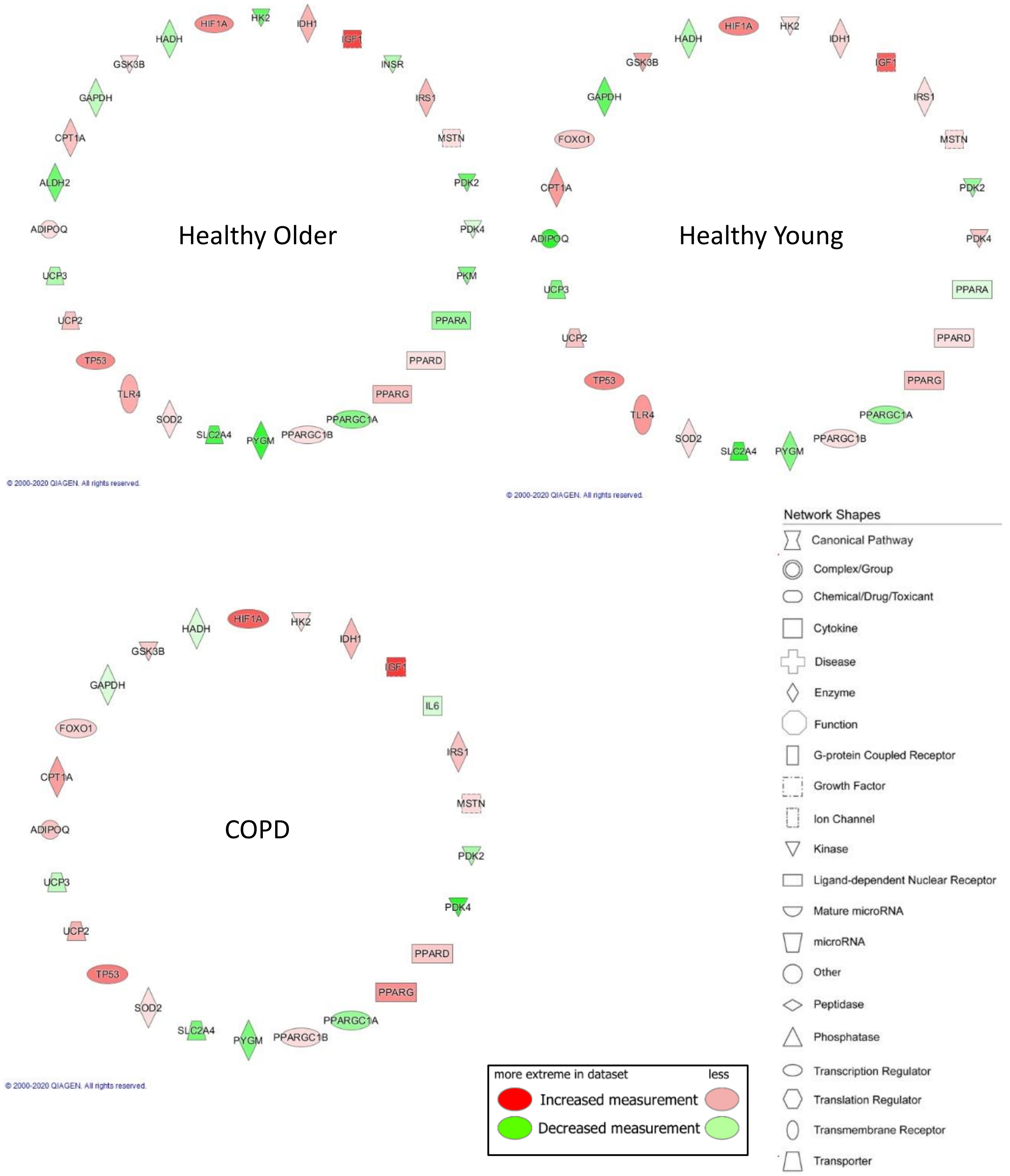

Figure S4. Differentially regulated muscle mRNAs associated with carbohydrate metabolism following 4 weeks exercise withdrawal compared to baseline in healthy older, healthy young and COPD groups. Abbreviated gene names are defined in Table S2.

REFERENCES

S1. Guidelines for the measurement of respiratory function. Recommendations of the British Thoracic Society and the Association of Respiratory Technicians and Physiologists. *Respiratory medicine* 1994; 88: 165-194.

S2. American Thoracic S, American College of Chest P. ATS/ACCP Statement on cardiopulmonary exercise testing. *Am J Respir Crit Care Med* 2003; 167: 211-277.

S3. Carter R, Peavler M, Zinkgraf S, Williams J, Fields S. Predicting maximal exercise ventilation in patients with chronic obstructive pulmonary disease. *Chest* 1987; 92: 253-259.

S4. Hayot M, Michaud A, Koechlin C, Caron MA, Leblanc P, Prefaut C, Maltais F. Skeletal muscle microbiopsy: a validation study of a minimally invasive technique. *Eur Respir J* 2005; 25: 431-440.

S5. Wibom R, Hagenfeldt L, von Dobeln U. Measurement of ATP production and respiratory chain enzyme activities in mitochondria isolated from small muscle biopsy samples. *Anal Biochem* 2002; 311: 139-151.

S6. Srere PA. [1] Citrate synthase: [EC 4.1.3.7. Citrate oxaloacetate-lyase (CoA-acetylating)]. Methods in Enzymology: Academic Press; 1969. p. 3-11.

S7. Constantin D, Constantin-Teodosiu D, Layfield R, Tsintzas K, Bennett AJ, Greenhaff PL. PPARdelta agonism induces a change in fuel metabolism and activation of an atrophy programme, but does not impair mitochondrial function in rat skeletal muscle. *The Journal of physiology* 2007; 583: 381-390.

S8. Murton AJ, Billeter R, Stephens FB, Des Etages SG, Graber F, Hill RJ, Marimuthu K, Greenhaff PL. Transient transcriptional events in human skeletal muscle at the outset of concentric resistance exercise training. *Journal of applied physiology (Bethesda, Md : 1985)* 2014; 116: 113-125.

S9. Murton AJ, Marimuthu K, Mallinson JE, Selby AL, Smith K, Rennie MJ, Greenhaff PL. Obesity Appears to Be Associated With Altered Muscle Protein Synthetic and Breakdown Responses to Increased Nutrient Delivery in Older Men, but Not Reduced Muscle Mass or Contractile Function. *Diabetes* 2015; 64: 3160-3171.

S10. Stephens FB, Wall BT, Marimuthu K, Shannon CE, Constantin-Teodosiu D, Macdonald IA, Greenhaff PL. Skeletal muscle carnitine loading increases energy expenditure, modulates fuel metabolism gene networks, and prevents body fat accumulation in humans. *The Journal of physiology* 2013.

S11. Porter C, Constantin-Teodosiu D, Constantin D, Leighton B, Poucher SM, Greenhaff PL. Muscle carnitine availability plays a central role in regulating fuel metabolism in the rodent. *The Journal of physiology* 2017; 595: 5765-5780.

S12. Constantin-Teodosiu D, Carlin JI, Cederblad G, Harris RC, Hultman E. Acetyl group accumulation and pyruvate dehydrogenase activity in human muscle during incremental exercise. *Acta Physiol Scand* 1991; 143: 367-372.

S13. van Loon LJ, Greenhaff PL, Constantin-Teodosiu D, Saris WH, Wagenmakers AJ. The effects of increasing exercise intensity on muscle fuel utilisation in humans. *The Journal of physiology* 2001; 536: 295-304.

S14. Bárány P, Wibom R, Hultman E, Bergström J. ATP production in isolated muscle mitochondria from haemodialysis patients: effects of correction of anaemia with erythropoietin. *Clinical Science* 1991; 81: 645-653.
