# Supplementary material for: Whole-body and muscle responses to aerobic exercise training and withdrawal in ageing and COPD
Source: Eur Respir J. 2022 May 12;59(5):2101507. doi: 10.1183/13993003.01507-2021 (PMC9095946; doi:10.1183/13993003.01507-2021)
Supplement: Supplementary file 2 [file ERJ-01507-2021.Shareable.pdf]

EUROPEAN RESPIRATORY JOURNAL
ORIGINAL RESEARCH ARTICLE
L.E. LATIMER ET AL.

SHAREABLE PDF

# Whole-body and muscle responses to aerobic exercise training and withdrawal in ageing and COPD

Lorna E. Latimer[1,2,8], Dumitru Constantin-Teodosiu[3,8], Bhavesh Popat[1,4], Despina Constantin[3,5], Linzy Houchen-Wolloff[1,2,6], Charlotte E. Bolton [3,5,7], Michael C. Steiner[1,2] and Paul L. Greenhaff[3,5]

[1]Dept of Respiratory Sciences, University of Leicester, Leicester, UK. [2]Institute for Lung Health, National Institute for Health Research Leicester Biomedical Research Centre – Respiratory, Glenfield Hospital, Leicester, UK. [3]MRC-Versus Arthritis Centre for Musculoskeletal Ageing Research, Division of Physiology, Pharmacology and Neuroscience, School of Life Sciences, University of Nottingham, Nottingham, UK. [4]University Hospitals of Derby and Burton NHS Foundation Trust, Derby, UK. [5]National Institute for Health Research Nottingham Biomedical Research Centre, Nottingham University Hospitals NHS Trust, Nottingham, UK. [6]University Hospitals of Leicester NHS Trust, Centre for Exercise and Rehabilitation Science, Glenfield Hospital, Leicester, UK. [7]Centre for Respiratory Research, Translational Medical Sciences, School of Medicine, University of Nottingham, City Hospital, Nottingham, UK. [8]Joint first authorship.

Corresponding author: Paul L. Greenhaff (paul.greenhaff@nottingham.ac.uk)

Check for updates

Shareable abstract (@ERSpublications)
**Muscle mitochondrial function is not impaired in age or COPD. Whole-body and mitochondrial exercise training adaptations are robust in young, evident in older and deficient in COPD. COPD adaptation may require high-intensity exercise training programmes.** https://bit.ly/3oe0k0S

**Cite this article as:** Latimer LE, Constantin-Teodosiu D, Popat B, *et al*. Whole-body and muscle responses to aerobic exercise training and withdrawal in ageing and COPD. *Eur Respir J* 2022; 59: 2101507 [DOI: 10.1183/13993003.01507-2021].

This single-page version can be shared freely online.

Copyright ©The authors 2022.

This version is distributed under the terms of the Creative Commons Attribution Licence 4.0.

Received: 27 May 2021
Accepted: 19 Sept 2021

## Abstract

***Background*** Chronic obstructive pulmonary disease (COPD) patients exhibit lower peak oxygen uptake ($V'_{O_2peak}$), altered muscle metabolism and impaired exercise tolerance compared with age-matched controls. Whether these traits reflect muscle-level deconditioning (impacted by ventilatory constraints) and/or dysfunction in mitochondrial ATP production capacity is debated. By studying aerobic exercise training (AET) at a matched relative intensity and subsequent exercise withdrawal period we aimed to elucidate the whole-body and muscle mitochondrial responsiveness of healthy young (HY), healthy older (HO) and COPD volunteers to whole-body exercise.

***Methods*** HY (n=10), HO (n=10) and COPD (n=20) volunteers were studied before and after 8 weeks of AET (65% $V'_{O_2peak}$) and after 4 weeks of exercise withdrawal. $V'_{O_2peak}$, muscle maximal mitochondrial ATP production rate (MAPR), mitochondrial content, mitochondrial DNA (mtDNA) copy number and abundance of 59 targeted fuel metabolism mRNAs were determined at all time-points.

***Results*** Muscle MAPR (normalised for mitochondrial content) was not different for any substrate combination in HO, HY and COPD at baseline, but mtDNA copy number relative to a nuclear-encoded housekeeping gene (mean±SD) was greater in HY (804±67) than in HO (631±69; p=0.041). AET increased $V'_{O_2peak}$ in HO (17%; p=0.002) and HY (21%; p<0.001), but not COPD (p=0.603). Muscle MAPR for palmitate increased with training in HO (57%; p=0.041) and HY (56%; p=0.003), and decreased with exercise withdrawal in HO (−45%; p=0.036) and HY (−30%; p=0.016), but was unchanged in COPD (p=0.594). mtDNA copy number increased with AET in HY (66%; p=0.001), but not HO (p=0.081) or COPD (p=0.132). The observed changes in muscle mRNA abundance were similar in all groups after AET and exercise withdrawal.

***Conclusions*** Intrinsic mitochondrial function was not impaired by ageing or COPD in the untrained state. Whole-body and muscle mitochondrial responses to AET were robust in HY, evident in HO, but deficient in COPD. All groups showed robust muscle mRNA responses. Higher relative exercise intensities during whole-body training may be needed to maximise whole-body and muscle mitochondrial adaptation in COPD.
